# ‘No Foot, No Future’: Lived Experiences of Foot Health and Care Access Among Rough‐Sleeping Adults in a UK Coastal Community—A Qualitative Study

**DOI:** 10.1002/jfa2.70145

**Published:** 2026-03-18

**Authors:** Joanna Bower, Joanne Paton

**Affiliations:** ^1^ University of Plymouth Plymouth UK

**Keywords:** access to care, foot care, health inequity, homelessness, podiatry, public health, qualitative research

## Abstract

**Introduction:**

People experiencing homelessness face profound barriers in accessing healthcare, particularly preventive services such as foot care. Globally, rough sleepers are vulnerable to foot‐related morbidity due to prolonged exposure, inadequate footwear and poor hygiene access. Despite this, foot health remains an under‐researched aspect of homelessness.

**Objective:**

To explore the lived experiences of rough‐sleeping adults in the United Kingdom in relation to foot health, self‐care practices and access to podiatric services, and to understand how these experiences influence their overall health and well‐being.

**Methods:**

This qualitative study employed interpretative phenomenological analysis (IPA) to investigate the narratives of seven adults (five men and two women, aged between 30 and 62, all identifying as White British) who were sleeping rough in an urban UK setting. Semistructured interviews were conducted at a community drop‐in centre. The consequent transcripts were analyzed thematically, focusing on participants' interpretations of foot health, access to care and coping within the context of homelessness.

**Results:**

Four themes were identified: foot care as survival, normalisation of foot health problems, barriers to foot care access and the charity sector as a lifeline. Participants reported widespread foot problems, including chronic pain, infections and poor nail health. Barriers to care included stigma, distrust of medical professionals, inflexible services and competing survival priorities. Lack of access to basic hygiene and footwear exacerbated foot health issues. In contrast, charitable services offering podiatry were described as vital sources of support and dignity.

**Conclusion:**

Foot health is integral to the mobility, independence and psychosocial well‐being of people experiencing homelessness. Findings highlight the need for flexible, outreach‐oriented and stigma‐informed approaches to podiatric care. Charitable models offer transferable insights for statutory and community services seeking to improve equitable access to foot care for socially marginalised populations.

## 1 Introduction

Homelessness continues to be a pressing public health concern worldwide, with individuals experiencing unstable housing often disproportionately burdened by physical, psychological and social health problems [1]. Among those most at risk are people who sleep rough. Individuals who live in public spaces, temporary shelters, abandoned buildings or vehicles, often without consistent access to hygiene facilities, healthcare or shelter from the elements [2]. Rough sleeping in England is increasing, with an estimated 4667 adults sleeping rough on a single night (Autumn 2024), representing a 20% rise from the previous year [3, 4]. Recorded numbers are now 91% higher than the three years previously and more than double those recorded in 2010. Women represent a growing proportion of the rough‐sleeping population, with a 20% increase reported in 2024, whereas UK nationals account for the majority of individuals affected (63%) [3]. These figures are likely to underestimate the true scale of rough sleeping as people in hidden locations and women avoiding visibility for safety reasons are frequently undercounted [5].

Rough sleeping exposes individuals to extreme weather conditions, injury and infection and can make self‐care difficult. These circumstances place a significant burden on foot health, which is essential not only for basic mobility but also for accessing food, shelter and services [4]. Previous studies have shown that homeless adults are at increased risk of foot‐related conditions such as ulcers, fungal infections and musculoskeletal problems. These conditions can escalate rapidly without timely intervention, potentially resulting in avoidable hospitalisations or long‐term disability [6, 7].

Despite the identified risks, foot health within the homeless population remains an overlooked dimension of care in both research and practice. In the United Kingdom, access to podiatry services for people experiencing homelessness is limited and inconsistent, with care most provided through charitable outreach services rather than routine statutory provision [8]. Mainstream NHS podiatry services are frequently restricted to individuals who meet specific eligibility criteria, such as the presence of diabetes or severe vascular disease, thereby excluding many homeless adults with significant but nonqualifying foot pathology, including chronic skin conditions, biomechanical pain or untreated infections. Additionally, requirements related to fixed addresses, appointment‐based systems or referral pathways through primary care further constrain access for those with unstable housing or limited engagement with general practice [9].

Where foot care is available, it is often episodic, under‐resourced and dependent on short‐term funding streams, resulting in inconsistent continuity of care [6]. Charitable foot care services may focus on immediate symptom relief, such as nail care, wound management or footwear provision, but are frequently unable to offer ongoing monitoring, preventative interventions or referral into specialist services [9]. Consequently, foot health needs may remain unmet until conditions deteriorate, increasing the likelihood of emergency department attendance or avoidable complications [10].

Existing literature on healthcare access among homeless populations tends to focus on emergency services or general medical care, with limited attention to podiatry or lower‐limb health. Studies from Canada, the United States and Australia have described systemic barriers such as cost, lack of insurance or stigma [6, 11, 12]. However, these studies also highlight that even in publicly funded health systems, structural barriers—such as inflexible service models and limited integration between health and social care—continue to impede equitable access to foot care for homeless adults. There is a paucity of research specifically exploring how rough sleepers themselves understand and experience foot care in their daily lives. Particularly in the context of public health systems such as the UK's National Health Service (NHS), which, despite apparent universal coverage, is not always accessible for marginalised populations [13, 14, 15].

This study aims to address that gap by exploring the lived experiences of adults (both men and women), currently sleeping rough in a UK city. Using a qualitative person‐centred approach, it investigates how foot health is perceived and managed in the context of homelessness, the challenges individuals face when trying to access care and the broader implications for well‐being and mobility. Understanding these experiences is essential for developing more inclusive and responsive footcare services, both within the United Kingdom and in international settings with similar public or mixed healthcare models.

## 2 Methods

### 2.1 Study Design

This study employed interpretative phenomenological analysis (IPA) to explore how individuals experiencing rough sleeping make sense of foot health and access to care within the context of daily survival. IPA is particularly appropriate for this topic as it enables in‐depth exploration of complex, embodied, and often marginalised experiences that are closely tied to identity, dignity and meaning. Grounded in phenomenology, hermeneutics and ideography, IPA prioritises detailed individual accounts over generalisability, allowing nuanced interpretation of how participants understand and negotiate foot health challenges within structural and social constraints [16].

### 2.2 Setting and Participants

The study was conducted in a mid‐sized UK coastal city with a documented rough‐sleeping population. Recruitment took place through a local homeless drop‐in centre providing basic support services. Participants were approached face‐to‐face to request participation in interviews by the designated gatekeeper (a volunteer from the charity,’ Plymouth Soup Run’, who had access to the rough sleeping community). Purposive sampling was used to select participants who met the following inclusion criteria [16]:

- Aged 18 years or older
- Currently experiencing rough sleeping (i.e., unsheltered and street homeless)
- Able to communicate in English
- Capable of providing informed consent

Individuals who were temporarily housed, in institutional care, or unable to provide informed consent were excluded.

Seven participants were interviewed to strike a balance between providing detailed accounts of individual experiences and having sufficient data to analyze similarities and differences between participants. No participants dropped out of the study. This sample size aligns with IPA methodological guidance, which emphasises depth of analysis over breadth [16].

### 2.3 Data Collection

A single semistructured interview was conducted in a private space within the drop‐in centre, involving the interviewer and interviewee only. An interview guide was developed based on a review of existing literature and refined through pilot testing with a volunteer familiar with rough sleeping. Interviews explored topics such as footcare routines, barriers to hygiene, footwear access, experiences of seeking help and perceptions of healthcare services. Participants were also invited to speak freely about their broader health and daily life challenges. Table 1 provides examples of the open‐ended questions used during the interviews.

**TABLE 1:** Sample of interview questions.

Question	Can you tell me about your experiences with foot health whilst living on the streets, and what these experiences have meant for you day to day?
Question	What has your experience been of getting help with your feet, and what affects whether you seek care or not?
Question	Can you describe your experiences with charitable or outreach foot care services, and what impact, if any, these have had on your daily life or sense of well‐being?

*Note:* The open‐ended questions allowed for follow‐up discussions depending on the responses from the interviewees.

All interviews were conducted by the lead researcher (JB) and digitally recorded with consent. The lead researcher (JB) is a female lecturer and podiatrist, trained in qualitative research. JB is one of the volunteer podiatrists visiting the drop‐in centre, working with the charity, Forgotten Feet. Interviews lasted between 15 and 40 min (mean: 27 min). Reflexive field notes were recorded within 24 h of each session to capture contextual details, initial interpretations and nonverbal cues.

### 2.4 Ethical Considerations

Ethical approval was obtained from the host university's research ethics committee (University of Plymouth‐ 4658). Informed consent was obtained from the participants verbally and in writing prior to participation. All participants were assigned pseudonyms to ensure confidentiality. Given the transient nature of rough sleepers, member checking was not feasible; however, feedback on preliminary findings was sought from volunteers at the drop‐in centre and charity, ‘Plymouth Soup Run’, to enhance the trustworthiness of interpretations.

### 2.5 Data Analysis

Interviews were transcribed verbatim and analyzed manually using IPA procedures [17]. The lead researcher (JB) read and re‐read each transcript to achieve immersion in the data, followed by line‐by‐line coding to identify significant statements and emergent themes. Transcripts, themes and codes were reviewed and checked for accuracy by the experienced qualitative researcher and co‐author (JP). A double hermeneutic process was used: participants made sense of their own experiences, and the researcher interpreted those interpretations. Codes were clustered into themes within and across cases to identify patterns whilst preserving idiographic nuance [16]. This iterative process involved multiple rounds of coding and theme refinement, with regular discussions between researchers JB and JP to address inconsistencies and refine the themes [18].

Reflexivity was maintained through the use of a reflective research journal, in which the lead researcher (JB) documented assumptions, evolving interpretations, emotional responses and potential biases throughout data collection and analysis. This reflexive process supported ongoing critical self‐awareness and helped to mitigate the influence of the researcher's professional background on data interpretation, thereby enhancing analytic credibility. Analytical rigour was further strengthened through supervisory review of a purposive sample of anonymised transcripts and associated preliminary codes. This process involved independent reading by an experienced qualitative supervisor (JP), followed by discussion of coding decisions, theme development and interpretive assumptions, supporting consistency and reflexive challenge rather than inter‐coder reliability. Credibility was additionally supported through prolonged engagement with the data, iterative coding and constant comparison across transcripts to ensure that themes were grounded in participants' accounts. Transferability was addressed through the provision of descriptions of the study setting, participant characteristics and analytic process, enabling readers to assess the applicability of findings to other contexts. The study adhered to the Consolidated Criteria for Reporting Qualitative Research (COREQ) checklist [18], ensuring transparency and comprehensive reporting of methodological and analytic decisions.

## 3 Results

Seven participants (five men and two women; aged 30–62) were interviewed. This sample size aligns with IPA methodological guidance, which emphasises depth of analysis over breadth [16]. Table 2 provides demographic and health‐related characteristics of the participants, including a history of foot problems and comorbidities such as diabetes, chronic obstructive pulmonary disease (COPD) and mental health conditions.

**TABLE 2:** Summary of participant characteristic**s**.

Participant	Gender	Age	Reported foot problems	Chronic illness	History of drug/alcohol use	Housing status	Notes
Anne	Female	38	Yes IGTN painful right forefoot	Asthma and mental health	Current drug user	Street homeless	Estranged from family; long‐term rough sleeper
Bob	Male	45	Yes tinea pedis	ADHD and PTSD	Current drug user and ex‐prison	Street homeless	High trust in charity sector; wary of institutions
Colin	Male	62	Yes left foot ankle sprain	COPD and restless legs	Former user	Lives in vehicle	Proud of self‐care; limited hygiene access
David	Male	40	Yes peripheral neuropathy and ulceration	Mental health and chronic wounds	Current prescribed drug user	Street homeless	Barriers due to mental illness and stigma
Eve	Female	55	Yes heel fissures and cellulitis	Diabetes and anxiety	Alcohol user	Lives in van	Cares for dogs; declines housing to keep pets
Fred	Male	61	Yes pitted keratolysis	Osteoarthritis and chronic pain	Former alcohol user	Squatting on boat	Skeptical of services; trauma history
Gary	Male	30	Yes IGTN	Mental health	Past/current drug user	Lives in vehicle	Supported by mental health and pet care charities

*Note:* This table presents demographic and contextual information for the study participants. These details provide insight into the complex health and social circumstances influencing foot care access and engagement among individuals experiencing homelessness. All health conditions and substance use histories were self‐reported at the time of interview. Participant names are pseudonyms. Housing status reflects circumstances at the time of data collection.

Abbreviations: ADHD, attention deficit hyperactivity disorder; COPD, chronic obstructive pulmonary disease; IGTN, ingrowing toe nail; PTSD, post‐traumatic stress disorder.

**FIGURE 1:**
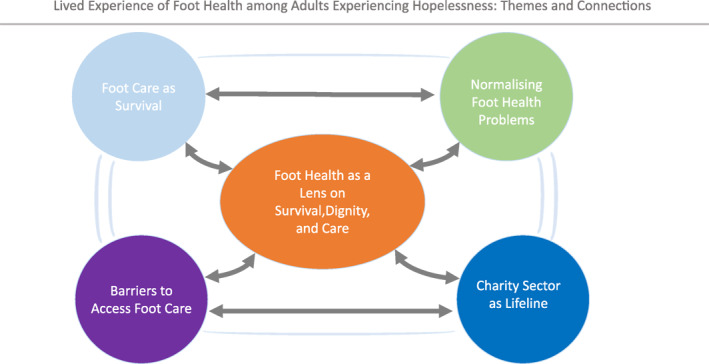
Conceptual diagram illustrating theme ‘foot health as lens on survival, dignity and care,’ linking the four superordinate themes identified through IPA analysis of participants' experiences.

All interviews were transcribed manually by the lead researcher (JB) using written notes and verbatim transcription, without the use of qualitative data analysis software. Thematic analysis was conducted through iterative reading and hand‐coding of transcripts, leading to the identification of four superordinate themes and one connecting theme. These reflected both shared and individual experiences of foot care needs, foot health challenges and access to services (Figure 1).

### 3.1 Integrated Findings and Discussion

This study explored the lived experiences of rough‐sleeping adults regarding foot health and access to care. Four key themes were identified: foot care as survival, normalising foot health problems, barriers to accessing foot care and the charity sector as a lifeline. The connecting theme, Foot Health as a Lens on Survival, Dignity and Care, highlighted how foot health is closely linked to participants' mobility, well‐being and overall quality of life.

These themes are discussed below, with participant narratives integrated alongside relevant literature to contextualise the findings.

#### 3.1.1 Foot Care as Survival

Participants consistently described their feet as central to daily survival, with mobility essential for accessing food, shelter and services. This was coded under Mobility Necessity and Functional Foot Health.

Bob emphasised this, saying:

They’re the tools of my trade. If I couldn’t walk, I’d be stuck.

Anne spoke of the importance of pain‐free feet

When my feet are sore, I just shut down

The metaphor of tools of the trade underscores the practical and existential importance of foot health, echoing findings that untreated foot conditions among homeless populations reduce mobility, increase injury risk and threaten independence [5].

Participants' narratives revealed how structural constraints—limited access to hygiene facilities, safe resting spaces or clean socks—impacted foot care practices.

Colin shared:

There’s no public taps. I use wet wipes to wash my feet.

David

My feet stink in summer when it's hot and there’s nought I can do about it … they stink in winter too

Here, the coding of Access Limitations shows that foot hygiene is not a matter of knowledge deficit but of constrained opportunity, a phenomenon widely reported in homelessness literature [15]. Survival priorities often meant foot care was deprioritised.

Gary explained:

I know how to look after them, I just don’t do it… priorities, innit?

Eve

I don’t think about me feet , what’s the point

Eve has lost access to her children and suffered numerous relationship breakdowns, including that with her mother. Her sadness pervaded throughout the interview. She appeared to be going through the motions of life, and her feet were a very low priority to her.

#### 3.1.2 Normalising Foot Health Problems

All participants described chronic foot issues—pain, infections and ingrown toenails—but these were frequently minimised or accepted as inevitable. Codes here included Chronicisation of Pain and Acceptance of Discomfort.

Eve noted:

No one’s ever asked me about my feet before,

David

I wouldn’t put you thought looking at my feet when they stink …not good it aint, I just go with it, its only pain

Fred

I know they are wrong but nothing can be done and so you just get on with it

This points to both invisibility and neglect in healthcare and research, consistent with global studies showing that chronic discomfort is normalised among homeless adults, particularly when overshadowed by more immediate concerns such as hunger or addiction [14, 15].

The psychological impact of untreated foot problems was also evident.

Bob said ‘When your feet hurt, it affects everything—your mood, how far you go, everything.’

This statement links the codes Physical–Emotional Interplay and Service Disengagement, aligning with evidence that unmanaged pain can exacerbate psychological distress and reduce engagement with healthcare [9, 18]. These findings emphasise that foot health is not merely a physical concern but is intertwined with mental well‐being and autonomy. Colin stated

It’s a real second‐class citizen system for homeless people … There’s an expectation that the patient is usually more needy and a druggy and I’m not, just want to be treated like others.

#### 3.1.3 Barriers to Accessing Foot Care

Participants described a range of systemic and personal barriers to accessing podiatric care, which were grouped into three interrelated sub‐themes: restrictive eligibility criteria, service inflexibility and bureaucratic burden and mistrust and emotional fatigue [19, 20, 21]. David expressed frustration with this approach, stating:

They won’t give me an appointment ‘cause I’m not diabetic.

This reflects a broader issue in healthcare systems where narrow eligibility criteria exclude those with complex overlapping vulnerabilities [22].

Participants highlighted inflexible service structures as a significant barrier to engagement, including long waiting times, limited referral pathways, appointment‐based systems and requirements for a permanent address. Navigating these systems was described as time‐consuming and emotionally draining, particularly when basic needs such as shelter and safety took precedence. Eve described the cumulative burden of these processes:

You have to prove you’re vulnerable enough… It’s exhausting.

Mistrust of healthcare providers was another recurring theme. Anne shared,

I don’t trust doctors. Not since back then. No one listens,

These sentiments are consistent with literature highlighting how past trauma, stigma and bureaucratic hurdles contribute to disengagement from formal healthcare [23, 24].

Gary

The drug therapy group I go to won't let me take the dog in. I stopped going there as she’s not waiting outside. I don’t go anywhere without her

Gary spoke in depth about the love he had for his dog, who is his ‘family’, and how attending health appointments was difficult *for him.*

I don’t worry about me feet, I am trying to get clean but I can’t

#### 3.1.4 The Charity Sector as a Lifeline

In contrast to statutory services, charitable organisations were described with warmth and gratitude. All participants praised the accessibility, empathy and practical support offered by these services. Eve described the podiatry service as follows:

a godsend,

Although Anne noted,

They give us shoes. Good ones. That matters.

Fred

The ladies give us time; it’s a real treat getting your feet done, you can't get this help in other places

These findings mirror international studies showing that community‐based outreach‐led services can bridge gaps in care and foster trust among marginalised populations [25, 26].

Charity‐run clinics were not only seen as providers of essential care but also as affirmations of dignity and compassion. For many, these services offered a rare sense of being valued and cared for—an important counterbalance to the dehumanising experiences often encountered in formal systems.

### 3.2 Implications for Practice and Policy

Findings from this study highlight the need for podiatry services to be delivered through flexible, low‐threshold models that reflect the realities of rough sleeping and housing instability. Participants described appointment‐based systems, referral requirements and eligibility criteria as significant barriers to care. In response, integrated care pathways could incorporate nonappointment‐based access, mobile or pop‐up clinics and walk‐in provision within existing outreach services. Such approaches align with NHS Inclusion Health guidance, which emphasises the adaptation of healthcare delivery models to reduce structural barriers faced by people experiencing homelessness and other socially excluded groups [27]. Embedding podiatry within multidisciplinary outreach teams may facilitate earlier intervention and reduce reliance on emergency care for preventable foot complications [28].

Routine foot health screening could also be integrated into outreach assessments for people experiencing homelessness, particularly for those with long‐term conditions such as diabetes, circulatory disease or neuropathy. Participants in this study reported significant foot problems that did not meet statutory thresholds for podiatry referral, resulting in delayed or absent care. Incorporating basic foot assessments into outreach encounters—alongside general health checks—could support early identification of risk, timely referral and preventative care. This approach is consistent with preventative health strategies and may help mitigate the progression of foot pathology among a population already at elevated risk of morbidity [1].

The findings further demonstrate the interconnected nature of foot health, mental health and substance use, with participants describing how psychological distress and addiction impacted both self‐care and engagement with services. Therefore, co‐locating podiatry services with mental health and substance use support may improve accessibility and continuity of care. Integrated models that address physical and psychosocial needs concurrently are likely to be more effective than isolated interventions, particularly for individuals who experience mistrust or fatigue in navigating fragmented services.

Finally, partnerships with third‐sector organisations and lived experience advocates are essential to enhancing service credibility and uptake. Participants consistently expressed greater trust in charitable services than in statutory provision, highlighting the importance of collaboration between NHS services and community organisations already embedded within homeless support networks. Formalising these partnerships may support more equitable service delivery whilst ensuring that podiatric care is culturally and contextually appropriate.

### 3.3 Strengths and Limitations

This study offers original insights into an under‐explored area of health inequality using a robust interpretative framework. The use of IPA enabled a rich exploration of individual narratives and meaning‐making. However, the small sample size and single‐site limit transferability. Furthermore, although feedback was sought from charity workers to support interpretation, participant member‐checking was not possible due to the transient nature of the population.

### 3.4 Future Research

Future research could look to explore the perspectives of healthcare providers, outreach workers and podiatrists to understand how structural and organisational factors shape service delivery. Comparative studies across regions or service models—statutory, charitable or hybrid—could clarify their impact on access, engagement and foot health outcomes.

Involving people with lived experience of homelessness in co‐design is essential. This could include advisory roles, peer researchers or participatory workshops to shape research questions, recruitment and intervention design. Participants in this study highlighted disengagement caused by inflexible services, mistrust, and a lack of understanding of homeless realities. Co‐design ensures interventions reflect these practical challenges, enhancing acceptability, accessibility and trust. It also promotes ethical inclusive research that empowers participants and grounds findings in real‐world experience.

## 4 Conclusion

This study highlights the critical importance of foot health in the daily lives of people experiencing rough sleeping. Although participants recognised the value of maintaining foot health, harsh living conditions and systemic barriers often made this impossible. Limited access to water, socks, footwear and health services left individuals with untreated foot problems, resulting in pain, reduced mobility and restricted engagement with support networks.

Charitable organisations emerged as a vital lifeline, providing accessible and respectful foot care that addressed unmet needs and fostered trust and visibility for a population often overlooked by mainstream services.

Improving foot health for homeless individuals requires approaches that extend beyond clinical settings. Services must be flexible, integrated with mental health and substance use support and developed in partnership with both statutory services and the third sector. Such efforts are essential to reduce inequities and ensure that care is accessible, responsive and ethically grounded.

## 5 Recommendations

- Embed foot health into broader homelessness outreach and assessment protocols.
- Offer walk‐in or mobile podiatry services tailored to people experiencing homelessness.
- Collaborate with trusted third‐sector organisations to increase access and build rapport.
- Train healthcare staff in trauma‐informed and stigma‐aware approaches to care delivery.
- Prioritise co‐production of services and research with people who have lived experience of homelessness.

## Author Contributions

**Joanna Bower:** conceptualisation, methodology, investigation, data curation, formal analysis, visualisation, writing – original draft, writing – review and editing, project administration. **Joanne Paton:** supervision, methodology, writing – review and editing, validation.

## Funding

This research was conducted as part of a postgraduate degree in Advanced Professional Practice at the University of Plymouth.

## Ethics Statement

This study received ethical approval from the University of Plymouth Ethics Committee (Ref: 4658). All participants provided informed consent prior to participation, and pseudonyms were used to protect their identities.

## Consent

Informed consent was obtained verbally and in writing from all participants. No identifying images or personal data are included in the manuscript.

## Conflicts of Interest

The authors declare no conflicts of interest.

## Data Availability

The data that support the findings of this study are available from the corresponding author upon request. The data are not publicly available due to privacy or ethical restrictions.
